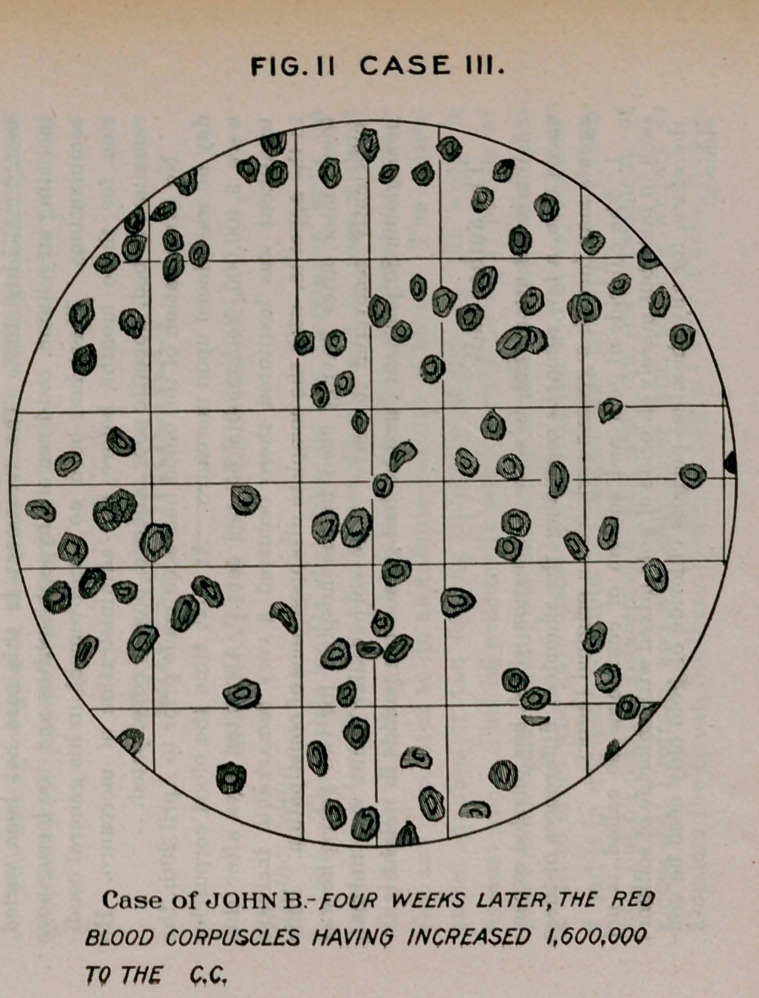# The Gold Combinations as Alteratives1Abstract of a paper read at the 21st Annual Meeting of the Mississippi Valley Medical Association, and published in the New York Medical Journal, November 23, 1895.

**Published:** 1896-01

**Authors:** Thomas Hunt Stucky

**Affiliations:** Professor of theory and practice and clinical medicine, Hospital College of Medicine, Louisville, Ky.


### Selection.

### THE GOLD COMBINATIONS AS ALTERATIVES.[1]

[Illustrated.]

By THOMAS HUNT STUCKY, M. D., Ph. D.,

Professor of theory and practice and clinical medicine, Hospital College of Medicine, Louisville, Ky.

UNDER the above title the author, in an elaborate paper, reports a series of cases tending to show wherein and how the gold combinations known as arsenauro and mercauro exert their peculiar effects. The observations were conducted during the spring and summer months, when the public wards of the hospital were generally free from acute diseases and all medicines were withdrawn except those under consideration.

Eight cases are reported in detail, from which we extract two, the second and third, as follows :

Case II.—F. P., aged 65 years, history of dissipation, admitted November, 1894 ; much jaundiced ; pain in right hypochondriac region ; pain and jaundice gradually disappeared, leaving him much emaciated ; anorexia ; bowels constipated ; diagnosis, cirrhosis of liver. Urine shows no marked deviation from health. Blood contains many small and large red cells, the red corpuscles numbering 3,253,000 ; hemoglobin, 52 per cent. Treatment, arsenauro, eight drops every four hours, hypodermically, commencing April 22d.

*May 5th.*—Patient appears to be stronger, remaining out of the bed and not requiring purgatives as formerly. Examination of the blood at

1. Abstract of a paper read at the 21st Annual Meeting of the Mississippi Valley Medical Association, and published in the *New York Medical Journal*, November 23, 1895.

this time shows 4,300,000 red corpuscles to the cubic millimeter; hemoglobin, 65 per cent.

*31st.*—While still using the gold combinations there was a diminution in the number to 3,850,000 and in hemoglobin to 60 per cent.

*June 19th.*—Patient seems to be in fairly good condition. During the past week he suffered from abdominal pain, diarrhea following this attack. Treatment continued.

*20th.*—Examination shows 4,650,000 red corpuscles; hemoglobin, 75 per cent.

While there have been fluctuations in the condition of the patient, he is after all much better as regards appetite and bodily vigor.

CASE III.—John B., teamster. Notes of this case began in 1893. He then had flattening, especially of the right side, diminished resonance, pain on pressure in supraclavicular region, nocturnal cough, muco-purulent expectoration. The tubercle bacilli could not be found, and many slides examined during the following two years failed to reveal their presence.

Changes in the physical signs have been slow, the area of dulness has extended to the right side, the heart is drawn to the right. The left lung presents the same signs as the right, but not so pronounced; he has constant fever, the evening rise usually 101° and not uncommonly reaching 103°. The treatment in this case has been varied, including strychnine, cod-liver oil. hypophosphites and the ferruginous combinations. There had been no improvement in his general condition for three months before the administration of mercauro. He remained in bed, appetite poor, anemic, bowels constipated.

No examination of the blood had been made prior to April 20th, the day he was placed upon mercauro. At that time the blood corpuscles were 3,400,000, hemoglobin 65 per cent. About ten days after this treatment was instituted there occurred a very remarkable increase in the appetite, with the complete disappearance of constipation. Four weeks later, after having been in the hospital for two years, he was sufficiently recovered to leave. The corpuscular count was normal, hemoglobin 80 per cent., and he had gained ten pounds in weight.

. . . . .

The author adds:

While not attempting to solve a question which has puzzled experienced men, a few remarks regarding the chemical differences of these agents may furnish a groundwork for an original theory.

1. The chloride of gold and sodium of commerce, so called, is not such in fact, but merely chloride of gold mixed with chloride of sodium; therefore, for any chemical purpose chloride of gold only need be considered.

2. Chloride of gold is an extremely unstable compound, its identity being readily destroyed by light or air, while the addition of the least amount of organic matter will almost instantly convert it into albuminate, which, upon contact with the mucous membrane or skin surface, (the albumin thus formed,) is extremely difficult of solution.

3. Gold bromide, even without the addition of the other material, is a more stable salt, is less sensitive to light, etc., and, when in combination with bromide of arsenic in aqueous solution as found in arsenauro and mercauro, this property of stability is increased to a seemingly very great extent.

4. This change in its attitude with reference to outside influences, from a chemical standpoint, may account for its altered therapeutic properties, and this may be said not only as regards the changes due to the combined therapeutic properties of the combination of gold and arsenic, but with reference solely to the probable modified or intensified quality, which appears to be a changed therapeutic equivalent in the gold itself.

5. As to what I conceive to be the reason of its changed or intensified therapeutic quality of gold in arsenauro and the like. The arsenic bromide added to this solution appears to have rendered the gold more tenacious of its dissolved condition, thus permitting it to be taken unaltered into the circulation.

The finding of gold in the urine after the administration of these solutions would appear to confirm this view.

## Figures and Tables

**Fig. I Case II. f1:**
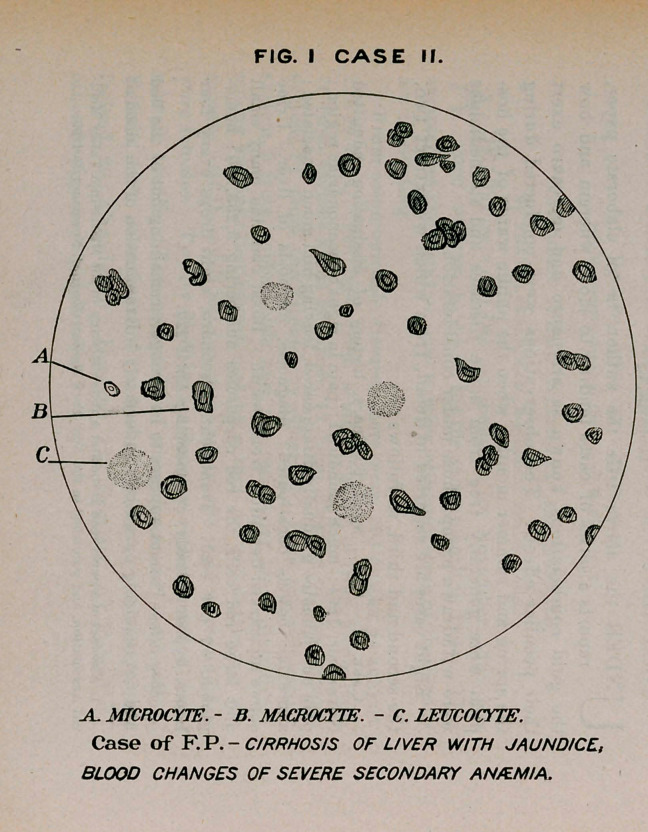


**Fig. II Case II. f2:**
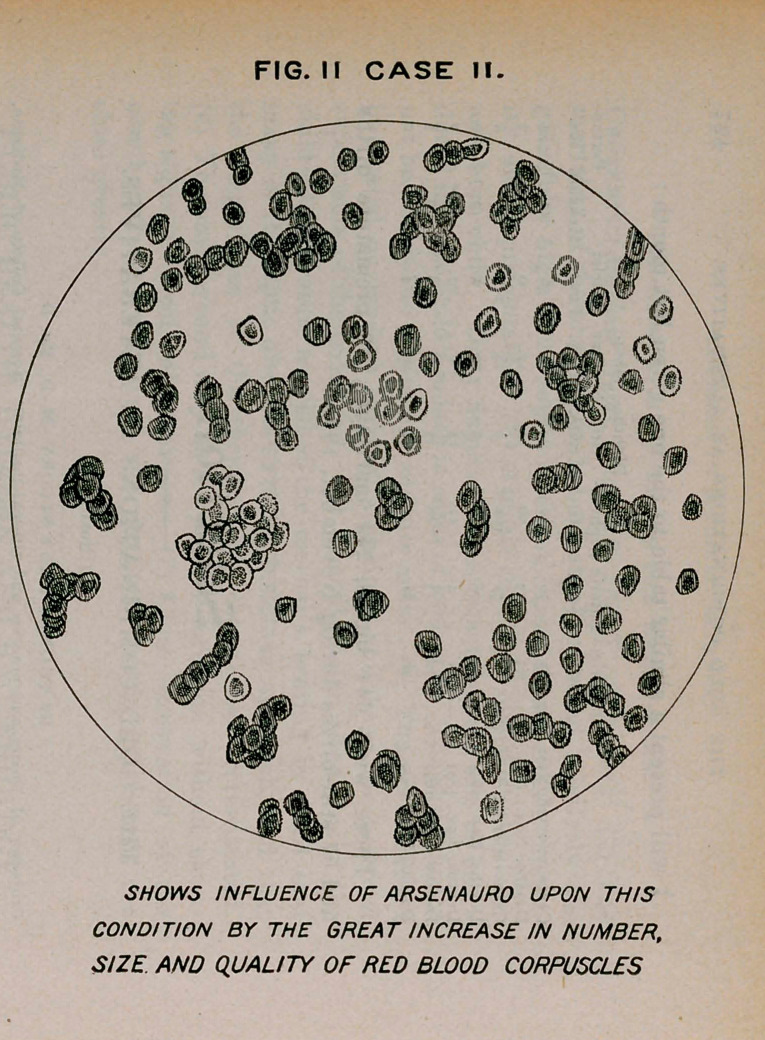


**Fig. I Case III. f3:**
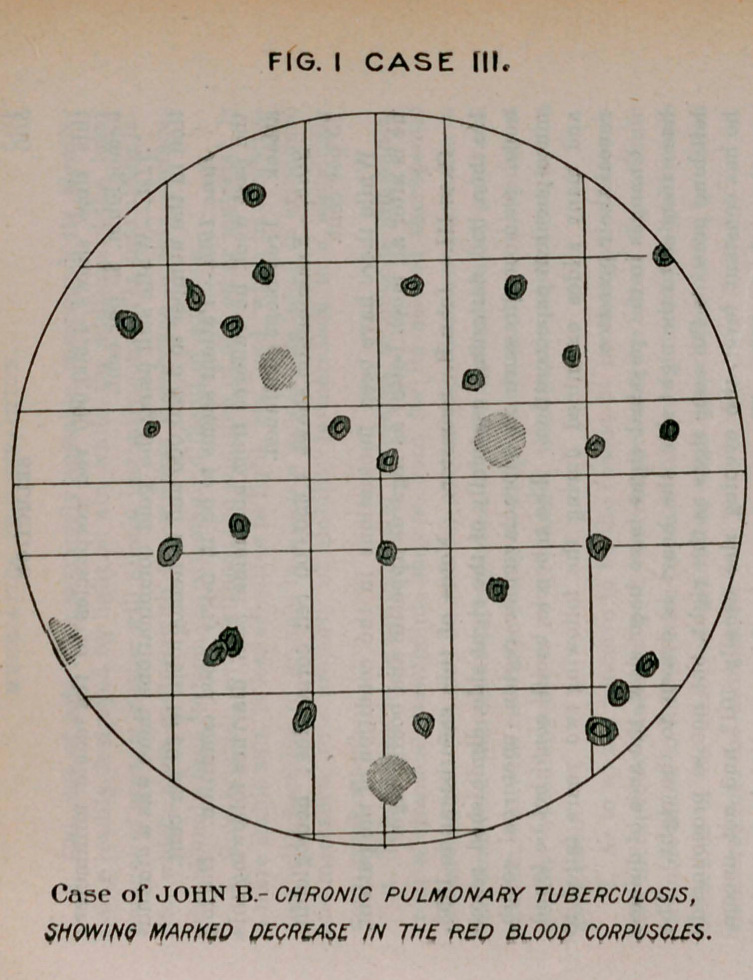


**Fig. II Case III. f4:**